# An efficient partial synthesis of 4′-*O*-methylquercetin via regioselective protection and alkylation of quercetin

**DOI:** 10.3762/bjoc.5.60

**Published:** 2009-11-04

**Authors:** Nian-Guang Li, Zhi-Hao Shi, Yu-Ping Tang, Jian-Ping Yang, Jin-Ao Duan

**Affiliations:** 1 Jiangsu Key Laboratory for TCM Formulae Research, Nanjing University of Chinese Medicine, 138 Xianlin Road, Nanjing, Jiangsu 210046, China, Tel/Fax: +86-25-85811916; 2 Department of Medicinal Chemistry, Nanjing University of Chinese Medicine, Nanjing, Jiangsu 210046, P.R. China; 3 Division of Organic Chemistry, China Pharmaceutical University, Nanjing, Jiangsu 211198, China

**Keywords:** alkylation, high yield, 4′-*O*-methylquercetin, partial synthesis, regioselective protection

## Abstract

An efficient partial 5-step synthesis of 4′-*O*-methylquercetin from quercetin in 63% yield is reported. This strategy relies on the selective protection of the catechol group with dichlorodiphenylmethane in diphenyl ether as solvent and on the selective protection of the hydroxyl groups at positions 3 and 7 with chloromethyl ether.

## Introduction

**Figure 1:**
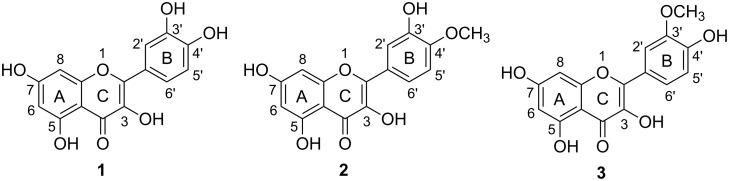
Structures of quercetin and methylated metabolites.

Flavonoids, such as flavones and flavonols, are secondary plant metabolites found in many foods, especially in fruits and vegetables [1–2]. Quercetin (**1**) (Figure 1), the major individual non-polymeric molecule among the polyphenols represents 60–75% of the flavonoid intake [3]. Quercetin is a very efficient antioxidant [4] and appears to be active against many diseases related to ageing such as cancer [5], cardiovascular [6] and neurodegenerative [7] diseases. In blood, quercetin is found mainly in metabolized forms. The non-degradative metabolism of quercetin involves three main modifications of the phenolic hydroxyl groups: methylation, sulfation and glucuronidation [2]. Plasma analysis of pigs fed with quercetin-rich diets show that quercetin is absent and only methylated metabolites such as 4′-*O*-methylquercetin (**2**, tamarixetin) and 3′-*O*-methylquercetin (**3**, isorhamnetin, Figure 1), mainly conjugated as glucuronides or sulfates [8], are present. With some variations in the relative abundance of the methylation positions, the same metabolism was also observed in rat [9] and in vivo cell cultures [10]. As the glucuronide or sulfate groups are readily deconjugated in tissues [11], these methylated metabolites are, presumably, the active molecules. Some studies using plasma samples show that the activities of the metabolites are rather different from those of quercetin itself [12].

These quercetin metabolites are not readily available commercially. Therefore, synthetic methods for the construction of these metabolites have become important in recent years. Liver microsomal preparation can be used [13–14] but this is not convenient for the preparation of larger quantities of the metabolites. Existing chemical syntheses of these metabolites [15–20] – where available – are either involved and/or low yielding. For example, 4′-*O*-methylquercetin (**2**, tamarixetin, Figure 1), one of the in vivo metabolites, was first synthesized by Jurd [15] from quercetin pentaacetate. The acetyl groups, which were attached to the flavone skeleton, were successively replaced by alkyl groups in the preferential order 7 > 4′ > 3 > 5 > 3′ to give **2** (along with many other methylquercetins) in consequently very low yield. Recently, Rolando’s group [18] obtained **2** in only 15% yield by sequential protection of the different phenolic functions of quercetin by using dichlorodiphenylmethane and benzyl bromide.

In this paper, a convenient and highly developed method is reported by using firstly dichlorodiphenylmethane in diphenyl ether to protect the hydroxy groups at positions 3′ and 4′ in quercetin (**1**). Then, chloromethyl ether is used to protect the hydroxy groups at positions 3 and 7 selectively in quercetin **4**, leaving the hydroxy group at position 5 unaffected. Thus, only five steps are needed to obtain 4′-*O*-methylquercetin (**2**, tamarixetin) in 63% yield.

## Results and Discussion

**Scheme 1:**
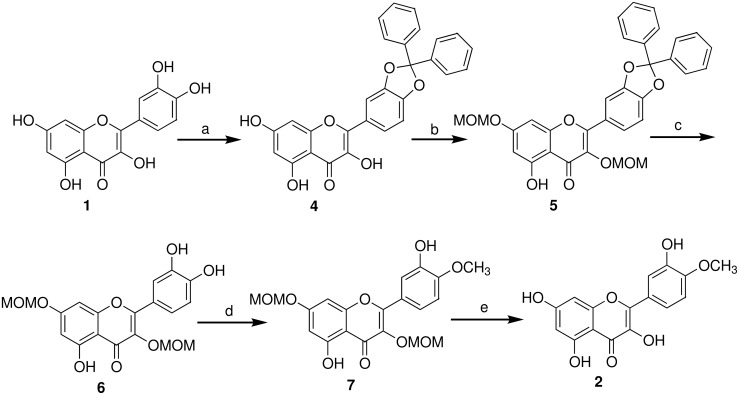
Synthesis of 4′-*O*-methylquercetin (**2**, tamarixetin). a) Ph_2_CCl_2_, Ph_2_O, 175 °C, 30 min, 86%; b) MOMCl, K_2_CO_3_, acetone, reflux, 6 h, 93%; c) Pd/C (10 wt %), H_2_ (1 atm), THF/EtOH, 8 h, 95%; d) MeI, K_2_CO_3_, DMF, 8 h, 92%; e) HCl (1.0 M) in Et_2_O/CH_2_Cl_2_ (1:1), 25 °C, 6 h, 90%.

4′-*O*-Methylquercetin (**2**, tamarixetin) is synthesized as shown in Scheme 1. This route is based on partial synthesis from quercetin (**1**) and relies on successive and selective protection of the different quercetin phenolic functions.

**Figure 2:**
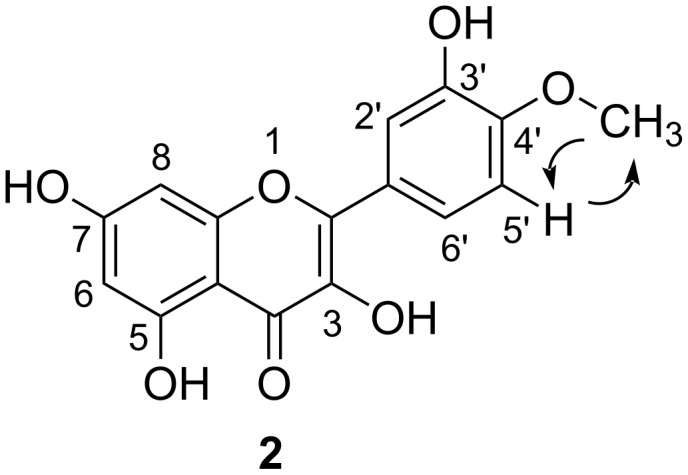
ROESY correlations of compound **2**.

Initially, we decided to adopt an alternative strategy which relies upon a selective protection of the catechol ring to make the later selective methylation easier. Hydroxy groups at position 3′ and 4′ in quercetin may be protected after chelation with borax [21]. However, the Wender group [22] and the Rolando group [23] reported that under these conditions quercetin methylation gave a complex mixture with at least three non-identified partially methylated quercetin ethers. So we decided to use the same strategy we developed for catechin based on the protection of the hydroxy groups at position 3′ and 4′ with dichlorodiphenylmethane in diphenyl ether [24]. In contrast to Jurd [25], who reported that quercetin protection with dichlorodiphenylmethane cannot be achieved directly and required initial protection of the 7-hydroxyl group, but in agreement with the recent paper by the Dangles group [26] and the Rolando group [18], we observed that diphenylmethylene is an efficient protecting group for the quercetin B ring vicinal hydroxyl groups. However, on following the latter method [18], the yield of the desired product **4** was very low when quercetin (**1**) was treated with 3 equiv of dichlorodiphenylmethane in the absence of solvent at 180 °C for 10 min. One reason might be that quercetin is a solid whilst dichlorodiphenylmethane is an oil and this could lead to mixing problems. We therefore applied the method developed by us [24] and treated **1** with 1.5 equiv of dichlorodiphenylmethane in diphenyl ether at 175 °C. The desired product **4** was obtained in 86% yield after a reaction time of only 30 min. We selected chloromethyl ether to protect the hydroxy groups at positions 3 and 7 in compound **4**. The deprotection conditions of methoxymethyl ether and diphenyl methyl groups are different, the methoxymethyl ether groups are labile under acidic conditions whilst the diphenylmethyl groups can be removed by hydrogenation. Treatment of **4** with an excess of chloromethyl ether (4 equiv) and K_2_CO_3_ (4.2 equiv) in acetone led to the formation of **5**, whose phenolic functions with the exception of the hydroxyl group at position 5 were protected. From compound **5**, we first focused on the catechol ring deprotection by cleaving the diphenylmethylene ketal. Two different methods were available for the cleavage of such diphenylmethylene ketals: hydrogenolysis or hydrolysis. Under hydrogenation conditions the ketal could be cleaved selectively. The best results were obtained with 10% palladium on carbon as the catalyst in THF/EtOH. TLC showed that the reaction was complete after 8 h. This method gave **6** in a 95% yield and no side products were detected by TLC analysis. This method is easy, efficient and fast. Treatment of **6** with 1.2 equiv of iodomethane led selectively to **7** with the desired methyl group in the 4′ position in 92% yield. The methyl group position was confirmed by Rotating Frame Overhauser Effect Spectroscopy (ROESY) (Figure 2). A cross-peak observed in the ROESY spectrum between δ 3.98 (C_4′_-OCH_3_) with 6.96 (C_5′_-H) confirmed that the position of the methyl group was at C_4′_-O (Figure 2). This corresponds to the one-dimensional (1D)-nuclear Overhauser effect (NOE) reported by the Rolando group [18].

The last step of the synthesis, the hydrolysis of the methyl ether group with hydrochloric acid, gave 4′-*O*-methylquercetin (**2**, tamarixetin) in 90% yield. The overall yield from **1** to **2** was 63%, which is much higher than during the previous procedure [18].

## Conclusion

In conclusion, we have succeeded in devising a partial synthesis of 4′-*O*-methylquercetin (tamarixetin) from quercetin in high yield. The strategy relies on the selective protection of the catechol group with dichlorodiphenylmethane at 175 °C using diphenyl ether as solvent and on the selective protection of the hydroxy groups at positions 3 and 7 with chloromethyl ether as well as the ability to remove the protecting groups under different reaction conditions. This reported protocol could be applied to the selective synthesis of other *O*-methylflavonoid isomers as well as to other flavonoid metabolites with other functions such as sulfate or glucuronide groups. These compounds will allow structure–activity relationship studies of flavonoids to be carried out. Their easily obtained labeled forms will give access to isotopic dilution dosage by LC-MS or LC-MS/MS and will help in the identification of unknown flavonoid metabolites.

## Experimental

### General

Reagents and solvents were purchased from commercial sources and used without further purification unless otherwise specified. Air- and moisture-sensitive liquids and solutions were transferred via syringe or stainless steel cannula. Organic solutions were concentrated by rotary evaporation below 45 °C at approximately 20 mm Hg. All non-aqueous reactions were carried out under anhydrous conditions using flame-dried glassware in an argon atmosphere in dry, freshly distilled solvents, unless otherwise noted. Yields refer to chromatographically and spectroscopically (^1^H NMR) homogeneous materials, unless otherwise stated. Reactions were monitored by thin-layer chromatography (TLC) carried out on 0.15–0.20 mm Yantai silica gel plates (RSGF 254) using UV light as the visualizing agent. Chromatography was performed on Qingdao silica gel (160–200 mesh) with petroleum ether (60–90) and ethyl acetate mixtures as eluant. Melting points (mp) were measured on a WRS-1B apparatus and were uncorrected. ^1^H NMR spectra were obtained with a Bruker AV-300 (300 MHz). Chemical shifts are recorded in ppm downfield from tetramethylsilane. *J* values are given in Hz. Abbreviations used are s (singlet), d (doublet), t (triplet), q (quartet), b (broad) and m (multiplet). ESI-MS spectra were recorded on a Waters Synapt HDMS spectrometer.

### 2-(2,2-diphenylbenzo[*d*][1,3]dioxol-5-yl)-3,5,7-trihydroxy-4*H*-chromen-4-one (**4**)

Dichlorodiphenylmethane (354 mg, 0.30 ml, 1.5 mmol) was added to a stirred mixture of quercetin (**1**) (302 mg, 1 mmol) in diphenyl ether (20 ml) and the reaction mixture was heated at 175 °C for 30 min. The mixture was cooled to room temperature and petroleum ether (50 ml) was added to give a solid compound. Then the solid was filtered and purified by column chromatography (25% ethyl acetate in petroleum ether) to yield **4** (400 mg, 86%) as a yellow solid [18]; mp 218–219 °C (lit. [18] 222–224 °C); ^1^H NMR (DMSO-*d*_6_, 300 MHz) δ 6.20 (d, *J* = 2.0 Hz, 1H, 6-H), 6.47 (d, *J* = 2.0 Hz, 1H, 8-H), 7.22 (d, *J* = 8.8 Hz, 1H, 5′-H), 7.46 (m, 6H, aromatic H), 7.58 (m, 4H, aromatic H), 7.79 (dd, *J* = 8.8, 1.8 Hz, 1H, 6′-H), 7.82 (d, *J* = 1.8 Hz, 1H, 2′-H), 9.61 (s, 1H, 3-OH), 10.81 (s, 1H, 7-OH), 12.37 (s, 1H, 5-OH); ESI-MS *m/z*: 467 [M+H]^+^, 489 [M+Na]^+^; Anal. calcd. for C_28_H_18_O_7_: C, 72.10; H, 3.89. Found: C, 72.18; H, 3.81.

### 2-(2,2-diphenylbenzo[*d*][1,3]dioxol-5-yl)-5-hydroxy-3,7-bis(methoxymethoxy)-4*H*-chromen-4-one (**5**)

Chloromethyl ether was added (1.28 ml, 16.84 mmol) to a stirred mixture of **4** (1.96 g, 4.21 mmol) and K_2_CO_3_ (2.45 g, 17.68 mmol) in dry acetone at room temperature. The reaction mixture was refluxed gently for 6 h. After cooling to room temperature, the reaction mixture was filtered. Removal of the solvent in vacuo followed by purification by column chromatography on silica gel of the residue with 20% ethyl acetate in petroleum ether afforded **5** (2.17 g, 93%) as a yellow solid; mp 102–104 °C; ^1^H NMR (CDCl_3_, 300 MHz) δ 3.21 (s, 3H, -OCH_3_), 3.48 (s, 3H, -OCH_3_), 5.16 (s, 2H, -OCH_2_O-), 5.22 (s, 2H, -OCH_2_O-), 6.45 (d, *J* = 2.2 Hz, 1H, 6-H), 6.59 (d, *J* = 2.2 Hz, 1H, 8-H), 6.98 (d, *J* = 8.2 Hz, 1H, 5′-H), 7.39 (m, 6H, aromatic H), 7.59 (m, 5H, aromatic H), 7.65 (dd, *J* = 8.2, 1.7 Hz, 1H, 6′-H), 12.51 (s, 1H, 5-OH); ESI-MS *m/z*: 555 [M+H]^+^, 477 [M+Na]^+^; Anal. calcd. for C_32_H_26_O_9_: C, 69.31; H, 4.73. Found: C, 69.38; H, 4.71.

### 2-(3,4-dihydroxyphenyl)-5-hydroxy-3,7-bis(methoxymethoxy)-4*H*-chromen-4-one (**6**)

To a solution of **5** (100 mg, 0.18 mmol) dissolved in ethanol (10 ml) and THF (10 ml) 10% Pd/C (2 mg) was added with vigorous stirring. Then the reaction vessel was evacuated and the atmosphere replaced with hydrogen. After 8 h, the reaction mixture was filtered through celite and the filtrate concentrated. The crude material was then chromatographed on silica gel (50% ethyl acetate in petroleum ether) to yield **6** (67 mg, 95%) as a yellow solid; mp 142–143 °C; ^1^H NMR (DMSO-*d*_6_, 300 MHz) δ 3.18 (s, 3H, -OCH_3_), 3.41 (s, 3H, -OCH_3_), 5.12 (s, 2H, -OCH_2_O-), 5.32 (s, 2H, -OCH_2_O-), 6.44 (d, *J* = 2.2 Hz, 1H, 6-H), 6.75 (d, *J* = 2.2 Hz, 1H, 8-H), 6.91 (d, *J* = 8.4 Hz, 1H, 5′-H), 7.47 (dd, *J* = 8.4, 2.4 Hz, 1H, 6′-H), 7.54 (d, *J* = 2.4 Hz, 1H, 2′-H), 9.34 (s, 1H, 3-OH), 9.76 (s, 1H, 7-OH), 12.59 (s, 1H, 5-OH); ESI-MS *m/z*: 391 [M+H]^+^, 413 [M+Na]^+^; Anal. calcd. for C_19_H_18_O_9_: C, 58.46; H, 4.65. Found: C, 58.49; H, 4.61.

### 5-hydroxy-2-(3-hydroxy-4-methoxyphenyl)-3,7-bis(methoxymethoxy)-4*H*-chromen-4-one (**7**)

Iodomethane (0.019 ml, 0.31 mmol) was added to a solution of **6** (100 mg, 0.26 mmol) in dry DMF (20 ml) K_2_CO_3_ (20 mg, 0.47 mmol) at room temperature. After 8 h, the reaction mixture was partitioned between 100 ml ethyl acetate and 100 ml water. The ethyl acetate layer was washed with brine, dried over MgSO_4_, filtered and concentrated. The crude material was purified by column chromatography (25% ethyl acetate in petroleum ether) to yield **7** (97 mg, 92%) as a yellow solid; mp 136–138 °C; ^1^H NMR (CDCl_3_, 300 MHz) δ 3.25 (s, 3H, -OCH_3_), 3.50 (s, 3H, -OCH_3_), 3.98 (s, 3H, -OCH_3_), 5.19 (s, 2H, -OCH_2_O-), 5.23 (s, 2H, -OCH_2_O-), 5.67 (s, 1H, 3′-OH), 6.45 (d, *J* = 2.1 Hz, 1H, 6-H), 6.61 (d, *J* = 2.1 Hz, 1H, 8-H), 6.96 (d, *J* = 9.2 Hz, 1H, 5′-H), 7.66 (dd, *J* = 9.2, 2.0 Hz, 1H, 6′-H), 7.68 (d, *J* = 2.0 Hz, 1H, 2′-H), 12.53 (s, 1H, 5-OH); ESI-MS *m/z*: 405 [M+H]^+^, 427 [M+Na]^+^; Anal. calcd. for C_20_H_20_O_9_: C, 59.40; H, 4.99. Found: C, 59.36; H, 5.01.

### 3,5,7-trihydroxy-2-(3-hydroxy-4-methoxyphenyl)-4*H*-chromen-4-one (**2**)

Hydrochloric acid (1 ml) was added to a stirred solution **7** (404 mg, 1 mmol) in CH_2_Cl_2_ (5 ml) and ether (5 ml) at 0 °C. The reaction mixture was allowed to warm to room temperature and stirred for a further 6 h. The reaction mixture was diluted with a large amount of ethyl acetate and washed with water and brine. The ethyl acetate layer was dried over MgSO_4_, filtered, then concentrated and the crude material purified by column chromatography (50% ethyl acetate in petroleum ether) to yield **2** (284 mg, 90%) as a yellow solid [18,27]; mp 253–255 °C (lit. [18] 252–254 °C, lit. [27] 253–256 °C); ^1^H NMR (DMSO-*d*_6_, 300 MHz) δ 3.85 (s, 3H, -OCH_3_), 6.19 (d, *J* = 2.0 Hz, 1H, 6-H), 6.42 (d, *J* = 2.0 Hz, 1H, 8-H), 7.07 (d, *J* = 8.6 Hz, 1H, 5′-H), 7.64 (dd, *J* = 8.6, 1.9 Hz, 1H, 6′-H), 7.67 (d, *J* = 1.9 Hz, 1H, 2′-H), 9.29 (s, 1H, 3-OH), 9.40 (s, 1H, 3′-OH), 10.76 (s, 1H, 7-OH), 12.44 (s, 1H, 5-OH); ESI-MS *m/z*: 317 [M+H]^+^, 339 [M+Na]^+^; Anal. calcd. for C_16_H_12_O_7_: C, 60.76; H, 3.82. Found: C, 60.71; H, 3.87.
